# Dosing of antidepressants in relation to body weight in children and adolescents with overweight

**DOI:** 10.1038/s41366-024-01677-2

**Published:** 2024-11-14

**Authors:** Julia Izsak, Elin E. Kimland, Jari Martikainen, Elin Dahlén, Jenny M. Kindblom

**Affiliations:** 1 Department of Drug Treatment, Region Västra Götaland, Sahlgrenska University Hospital, Gothenburg, Sweden; 2 Department of Internal Medicine and Clinical Nutrition, Institute of Medicine, The Sahlgrenska Academy, University of Gothenburg, Gothenburg, Sweden; 3 Swedish Medical Products Agency, Uppsala, Sweden; 4 Department of Pharmacy, Faculty of Pharmacy, Uppsala University, Uppsala, Sweden; 5 Bioinformatics and Data Centre, The Sahlgrenska Academy, University of Gothenburg, Gothenburg, Sweden; 6 Department of Clinical Science and Education, Södersjukhuset, Karolinska Institute, Stockholm, Sweden

**Keywords:** Epidemiology, Drug development

## Abstract

Overweight and obesity in children and adolescents may impact pharmacokinetics and drug exposure. The aim of the present study was to evaluate doses of antidepressants in relation to body weight in children. We used data from the BMI Epidemiology Study (BEST) Gothenburg cohort and the National Prescribed Drug Register and included children and adolescents with a prescription of fluoxetine (*n* = 347) or sertraline (*n* = 733) and a weight measurement. For fluoxetine, individuals with overweight or obesity received slightly lower doses at first prescriptions, but not in iterated prescriptions. The weight-normalized dose was lower in individuals with overweight or obesity in first and iterated prescriptions, compared with normal weight (*p* < 0.01). For sertraline, there were no significant dose differences between individuals with overweight or obesity, compared with normal weight. However, pronounced differences were seen in dose per kilogram body weight in both first and iterated prescriptions (*p* < 0.01). We conclude that the doses of fluoxetine and sertraline were essentially similar in individuals with overweight or obesity, but the weight-normalized doses were clearly lower. Given the ongoing obesity epidemic, larger studies addressing optimal dosing in individuals with elevated weight are warranted.

## Introduction

The use of antidepressant medications in children and adolescents has increased considerably over the last 10–15 years [1, 2]. In addition, childhood obesity rates have more than tripled since the 1970s [3]. The association between the increasing use of antidepressants and the obesity epidemic in children and adolescents is not fully understood, but there is likely a bidirectional relationship. Obesity appears to increase the risk of developing depression, and also, depression and the use of antidepressants may stimulate weight gain [4]. The most used class of antidepressants in the pediatric population are selective serotonin reuptake inhibitors (SSRIs). Fluoxetine, labeled for major depressive disorder to children from eight years of age, and sertraline, labeled for obsessive-compulsive disorder to children from six years of age, represent the most used SSRI substances among children in Sweden [2, 5].

Medications are commonly dosed according to weight or age in children to adjust for physiologic and pharmacokinetic differences compared to adults. Overweight and obesity add additional complexity to drug disposition, as pharmacokinetic alterations related to the higher proportion of fat mass, and potential changes in drug metabolism and clearance, might result in substantial differences in drug exposure [6]. However, recommendations regarding optimal dosing of SSRIs in children in relation to body weight are lacking [7–10]. In practice, the current dosing approach for SSRIs in children is to initiate treatment at a low starting dose and increase to a maintenance dose based on response and tolerability. The usual starting dose for fluoxetine ranges from 5 to 10 mg per day with a maintenance dose ranging between 20 to 60 mg per day [10, 11]. For sertraline, the starting dose is typically 12.5 to 25 mg daily and the maintenance dose falls usually between 50 and 200 mg [10, 11]. Both substances have clearly stated maximum doses. To our knowledge, no prior studies have evaluated dosing of SSRIs in relation to body weight in prescriptions to children and adolescents.

The aim of this study was to evaluate the prescribed doses of SSRIs in relation to body weight in a cohort of children and adolescents from the population-based BMI Epidemiology Study Gothenburg.

## Methods

The BEST Gothenburg cohort has developmental height and weight measurements available from school health care and child health care [12], and information on diagnoses and prescribed drugs from high-quality national registers in Sweden. Individuals in the BEST Gothenburg cohort were eligible for the present study if they had at least one prescription of fluoxetine or sertraline dispensed before 18 years of age in the National Prescribed Drug Register (initiated 2005, study follow up until January 31^st^, 2021), and in addition had a weight measurement available in the interval from 3 months before to 6 months after the antidepressant dispensing. The data included in the present study required that the prescriptions were dispensed by the pharmacy. For simplicity, the word “prescription” is used throughout the manuscript. Antidepressants were defined using the Anatomical Therapeutic Chemical (ATC) code N06AB03 for fluoxetine and N06AB06 for sertraline [13].

The BMI Epidemiology Study Gothenburg has been approved by the ethics committee of the University of Gothenburg (013-10).

### Exposure and background factors

The exposures in the present study were body weight, overweight and obesity status. We calculated Body Mass Index (BMI) as weight in kilograms divided by height in meters squared (kg/m^2^) and categorized the values according to the International Taskforce for Obesity´s age- and sex-specific cutoffs for overweight and obesity [14]. Furthermore, using information from the Swedish National Patient Register, we defined the presence of psychiatric (F00-F99) and neuropsychiatric disorders (F84 and F90-F98) before the prescription using the International Classification of Diseases (ICD) system. The medications were defined using the following ATC codes: N06BA and C02AC02 (treatment for ADHD), N05A (antipsychotics), N05B, R06AD (anxiolytics) and N05C (sedatives) in the National Prescribed Drug Register, dispensed within 6 months before, or 6 months after, the dispensing date of antidepressant.

### Outcomes

The National Prescribed Drug Register is held by the National Board of Health and Welfare in Sweden and was initiated in 2005. The outcomes in the present study were dose of the antidepressant medication (mg), and dose per kilogram body weight (mg/kg) for the first eligible prescription dispensed before January 31^st^, 2021. The included prescriptions could be first or iterated prescriptions. For individuals with more than one dispensed prescription that fulfilled the inclusion criteria, we included the first eligible prescription. To ascertain the dose, we used tablet strength and information given with the prescription available as free text in the National Prescribed Drug Register. Two of the investigators (JI and JMK) reviewed the information independently and retrieved the prescribed dose. For the weight-normalized dose, we used the dose divided by the subject´s body weight (mg/kg) in the interval from 3 months before to 6 months after the date of the antidepressant dispensing.

### Statistical analyses

Descriptive statistics for continuous variables are presented using mean and standard deviation (SD), and categorical variables using number and percentage. We used a two-sided Welch´s t-test to test the difference between groups.

## Results

In the present study we used the population-based cohort BMI Epidemiology Study Gothenburg to evaluate the prescription of antidepressants in relation to body weight in children and adolescents. In total, 1080 individuals below 18 years of age were prescribed either fluoxetine (*n* = 347) or sertraline (*n* = 733) at least once during the study period and had a weight measurement available in the required interval. The mean age at prescription was 14.1 years (standard deviation [SD] 2.6), and 55.0% were females. Overweight or obesity was seen in 24.8% of the individuals in the cohort. Fluoxetine was prescribed at an average dose of 16.7 mg (SD 6.21) for the first and 23.2 mg (9.5) for iterated prescriptions, while sertraline was prescribed at an average dose of 44.3 mg (17.8) for first and 69.1 mg (38.6) for iterated prescriptions (Table 1).

**Table 1:** Cohort descriptives.

Cohort descriptives	All Mean (SD)	Range	Without overweight or obesity mean (SD)	Overweight (including obesity) mean (SD)	Obesity mean (SD)
Age at prescription (years)	14.1 (2.6)	3.5–18.0	14.2 (2.5)	13.7 (2.7)	14.0 (2.8)
Height (cm)	162.1 (14.2)	102.0–201.0	162.1 (13.9)	161.9 (15.2)	164.3 (14.6)
Weight (kg)	55.7 (16.6)	16.0–125.0	50.9 (12.4)	70.2 (19.3)	86.4 (20.2)
BMI (kg/m^2^)	20.8 (4.3)	12.7–46.7	19.0 (2.5)	26.3 (4.2)	31.6 (4.4)
BMI Z-score	0.45 (1.2)	−2.9–3.8	−0.06 (0.9)	2.0 (0.5)	2.8 (0.4)
	***N*** **(%)**		***N*** **(%)**	***N*** **(%)**	***N*** **(%)**
Patients	1080 (100)	NA	812 (75.2)	268 (24.8)	63 (5.8)
Females	594 (55.0)	NA	467 (57.5)	127 (47.4)	30 (47.6)
First prescription	674 (62.4)	NA	524 (64.5)	150 (56.0)	31 (49.2)
Any psychiatric diagnosis before prescription	956 (88.5)	NA	716 (88.2)	240 (89.6)	57 (90.5)
Neuropsychiatric diagnosis	496 (45.9)	NA	346 (42.6)	150 (56.0)	38 (60.3)
Pharmacological treatment for ADHD	266 (24.6)	NA	182 (22.4)	84 (31.3)	22 (34.9)
Pharmacological treatment with antipsychotics	146 (13.5)	NA	101 (12.4)	45 (16.8)	9 (14.3)
Pharmacological treatment with anxiolytics	432 (40.0)	NA	325 (40.0)	107 (39.9)	25 (39.7)
Pharmacological treatment with sedatives	345 (31.9)	NA	246 (30.3)	99 (36.9)	20 (31.7)
**Prescription descriptives**	**Mean (SD)**	**Range**	**Mean (SD)**	**Mean (SD)**	**Mean (SD)**
Fluoxetine (*n* = 347)			*n* = 270	*n* = 77	*n* = 21
Prescribed dose (mg)	19.2 (8.3)	4.0–60.0	19.4 (7.7)	18.8 (10.0)	21.4 (12.0)
Prescribed dose per kg (mg/kg)	0.35 (0.16)	0.10–0.91	0.37 (0.16)	0.26 (0.12)	0.24 (0.12)
Sertraline (*n* = 733)			*n* = 542	*n* = 191	*n* = 42
Prescribed dose (mg)	53.6 (29.9)	10.0–300.0	52.9 (29.5)	55.9 (30.9)	52.4 (19.8)
Prescribed dose per kg (mg/kg)	1.04 (0.59)	0.16–4.92	1.11 (0.61)	0.84 (0.47)	0.65 (0.27)

Characteristics of the included individuals (*n* = 1080) from the BMI Epidemiology Study Gothenburg, with at least one dispensing of fluoxetine or sertraline below 18 years of age, and height and weight available in the interval from 3 months before to 6 months after the antidepressant dispensing. Overweight and obesity were defined according to the cutoff´s defined by the International Obesity Taskforce [14]. Register-data was collected regarding psychiatric diagnosis (F00-F99) and neuropsychiatric diagnosis (F84 or F90-F98) before the dispensing of antidepressant. Treatment for attention-deficit hyperactivity disorder (ADHD; N06BA and C02AC02), antipsychotics (N05A), anxiolytics (N05B, R06AD), sedatives (N05C) was considered in the interval from 6 months before to 6 months after the dispensing of antidepressants. Data is presented as means (standard deviation [SD]) and range for continuous variables, and as number (%) for categorical variables.

Overall, there were no significant differences in the fluoxetine dose between overweight and normal weight individuals when first and iterated prescriptions were considered together. When considered separately, individuals with overweight received slightly lower doses of fluoxetine in first prescriptions, but no significant differences were seen for iterated prescriptions. The weight-normalized dose was clearly lower in individuals with overweight or obesity in first and iterated prescriptions (Table 2). For sertraline, there were no significant differences between individuals with overweight compared with normal weight in first or iterated prescriptions. In contrast, pronounced differences were seen in weight-normalized dose in both first and iterated prescriptions (Table 2).

**Table 2:** Antidepressant dose and dose per kg body weight for first or iterated prescription in children with normal weight or overweight.

**Panel A**			
**Fluoxetine**	**Without overweight or obesity** ***Mean (SD)***	**Overweight (including obesity)** ***Mean (SD)***	**Obesity** ***Mean (SD)***
All prescriptions	*n* = 270	*n* = 77	*n* = 21
Dose (mg)	19.4 (7.7)	18.8 (10.0)	21.4 (12.0)
Dose per kg body weight (mg/kg)	0.37 (0.16)	0.26 (0.12)***	0.24 (0.12)***
First prescription	*n* = 166	*n* = 48	*n* = 10
Dose (mg)	17.3 (6.4)	15.0 (5.3)*	15.0 (5.3)
Dose per kg body weight (mg/kg)	0.34 (0.14)	0.22 (0.08)***	0.18 (0.05)***
Iterated prescription	*n* = 104	*n* = 29	*n* = 11
Dose (mg)	22.7 (8.5)	25.0 (12.5)	27.3 (13.5)
Dose per kg body weight (mg/kg)	0.42 (0.17)	0.33 (0.15)**	0.30 (0.14)*
**Sertraline**	**Without overweight or obesity** ***Mean (SD)***	**Overweight** ***Mean (SD)***	**Obesity** ***Mean (SD)***
All prescriptions	*n* = 542	*n* = 191	*n* = 42
Dose (mg)	52.9 (29.6)	55.9 (30.9)	52.4 (19.8)
Dose per kg body weight (mg/kg)	1.11 (0.61)	0.84 (0.47)***	0.65 (0.27)***
First prescription	*n* = 358	*n* = 102	*n* = 21
Dose (mg)	44.7 (18.2)	43.8 (16.7)	44.8 (9.6)
Dose per kg body weight (mg/kg)	0.97 (0.45)	0.69 (0.26)***	0.62 (0.19)***
Iterated prescription	*n* = 184	*n* = 89	*n* = 21
Dose (mg)	68.8 (39.3)	69.7 (37.0)	56.0 (26.1)
Dose per kg body weight (mg/kg)	1.39 (0.76)	1.00 (0.59)***	0.67 (0.34)***
**Panel B**			
**Fluoxetine**	**Boys (*n*** = **124)** ***Mean (SD)***	**Girls (*n*** = **223)** ***Mean (SD)***	
Dose (mg)	18.2 (8.5)	19.8 (8.0) NS	
Dose per kg body weight (mg/kg)	0.32 (0.16)	0.36 (0.15)*	
	**Without NP diagnosis (*n*** = **231)** ***Mean (SD)***	**With NP diagnosis (*n*** = **116)** ***Mean (SD)***	
Dose (mg)	19.6 (7.9)	18.6 (8.9) NS	
Dose per kg body weight (mg/kg)	0.35 (0.16)	0.34 (0.16) NS	
**Sertraline**	**Boys (*n*** = **362)** ***Mean (SD)***	**Girls (*n*** = **371)** ***Mean (SD)***	
Dose (mg)	51.7 (29.5)	55.5 (30.2) NS	
Dose per kg body weight (mg/kg)	1.01 (0.56)	1.07 (0.60) NS	
	**Without NP diagnosis (*n*** = **353)** ***Mean (SD)***	**With NP diagnosis (*n*** = **380)** ***Mean (SD)***	
Dose (mg)	55.6 (30.5)	51.8 (29.2) NS	
Dose per kg body weight (mg/kg)	1.02 (0.56)	1.06 (0.62) NS	

Panel A; Antidepressant dose and dose per kg body weight in relation to overweight or obesity according to the cutoff´s defined by the International Obesity Taskforce [14] for first, iterated and all prescriptions.

Panel B; Fluoxetine and sertraline dose and dose per kg body weight in relation to sex, neuro-psychiatric co-morbidities (defined as an F84 or F90-F98 diagnosis before the antidepressant prescription), for all prescriptions.

Values are presented as mean (standard deviation [SD]). Statistically significant differences compared with individuals without overweight or obesity, boys, and individuals without neuro-psychiatric diagnoses, respectively.

*SD* standard deviation, *NS* not significant, *NP* neuro-psychiatric.

***p < 0.001; **p < 0.01; *p < 0.05.

Boys and girls were prescribed similar doses, but girls were prescribed a slightly higher weight-normalized dose for fluoxetine, with no significant difference for sertraline (Table 2). Individuals with and without neuropsychiatric disorders received similar doses and weight-normalized dose (Table 2).

## Discussion

In the present study, we evaluated real-world data on prescribed doses of fluoxetine and sertraline in children and adolescents according to overweight (including obesity) and normal weight. We found that the prescribed dose was similar for individuals with overweight and obesity compared with normal weight. Yet, when normalized to body weight, both fluoxetine and sertraline were prescribed at significantly lower dose/kg in patients with overweight. This novel observation of lower weight-normalized dose of SSRIs in children and adolescents with overweight and obesity could potentially result in compromised treatment outcomes in this patient population, and therefore warrants further attention.

In a previous study by Mansoor et al. the relationship of BMI and SSRI concentrations in 296 adolescents (*n* = 48 with overweight and *n* = 90 with obesity) with treatment resistant depression was evaluated. The authors found lower SSRI concentrations (pooled data) in patients with higher BMI [15]. In a recent study using therapeutic drug monitoring, 78 children and adolescents treated with sertraline were included. The authors found that weight-adjusted doses associated more strongly with serum concentrations than those without weight adjustment, indicating an impact of high body weight on serum concentrations [16]. In contrast to the results of the aforementioned two studies, a report from Sweden evaluating serum concentrations after antidepressant exposure in children and adolescents found no association between BMI and serum concentrations of sertraline [17]. Thus, the current state of knowledge regarding the effect of BMI on blood concentrations of sertraline in children and adolescents is somewhat conflicting.

Regarding fluoxetine, both the Summary of Product Characteristics and a review article highlight the impact of body weight on steady-state plasma concentrations and recommend lower doses of fluoxetine for children with lower body weight [18, 19]. Importantly, dosing considerations for children and adolescents with overweight or obesity are not addressed [18, 19].

Our results show that pediatric patients with overweight and obesity are prescribed lower doses of fluoxetine and sertraline per kilogram of body weight. While SSRIs are typically titrated based on therapeutic response and adverse effects, they have clearly defined maximum doses to avoid overdose and toxicity, which may contribute to the lower per-kilogram doses observed in these patients. Interestingly, studies in adults suggest that individuals with higher BMI or weight may have a reduced response to antidepressants, including SSRI [20]. This reduced response may be linked to lower blood concentrations of the drugs, but other factors associated with overweight—such as inflammatory dysregulation, altered blood-brain barrier permeability, leptin resistance, and comorbid medical conditions—could also contribute to treatment resistance in these patients [20]. However, it remains unclear whether BMI or weight similarly affects treatment response to SSRIs in children and adolescents.

It is still uncertain whether patients with overweight or obesity would benefit from higher or lower doses of antidepressants, as studies have shown mixed results on how elevated body weight may affect SSRI blood concentrations and therapeutic outcomes in children and adolescents. Considering the potential risks of treatment failure or drug toxicity in this population, larger studies are needed to measure both drug concentrations and treatment outcomes related to dosing in children with and without overweight and obesity. These data will be crucial for optimizing and individualizing dosing strategies for children and adolescents with overweight and obesity.

## Conclusion

Our results indicate similar doses but significantly lower weight-normalized doses in SSRI prescriptions in pediatric patients with overweight and obesity.

Given the ongoing obesity epidemic, there is an urgent need for larger studies on dosing, exposure and treatment outcome in children and adolescents with overweight and obesity, treated with SSRI, for optimized treatment and a personalized medicine approach in the future.

## Data Availability

Research data are not publicly available due to privacy and ethical restrictions. However, anonymised data required to reproduce results can be made available from the corresponding author on reasonable request on approval from the University of Gothenburg, if the data can be made available according to mandatory national law.
